# The Effectiveness of the GnRH Agonist/Antagonist Protocols for Different Poseidon Subgroups of Poor Ovarian Responders

**DOI:** 10.3390/jcm14062026

**Published:** 2025-03-17

**Authors:** Jelena Havrljenko, Vesna Kopitovic, Aleksandra Trninic Pjevic, Stevan Milatovic, Sandro Kalember, Filip Katanic, Tatjana Pavlica, Nebojsa Andric, Kristina Pogrmic-Majkic

**Affiliations:** 1 Ferona Fertility Clinic, Sarplaninska 19, 21000 Novi Sad, Serbia; vukosavljevicj@yahoo.com (J.H.); vesna.kopitovic@yahoo.com (V.K.); alex.trninic.pjevic@gmail.com (A.T.P.); sandrokalember@yahoo.com (S.K.); katanic.f@gmail.com (F.K.); 2 Department of Biology and Ecology, Faculty of Sciences, University of Novi Sad, Trg Dositeja Obradovica 2, 21000 Novi Sad, Serbia; tatjana.pavlica@dbe.uns.ac.rs (T.P.); nebojsa.andric@dbe.uns.ac.rs (N.A.); 3 Faculty of Medicine, University of Novi Sad, Hajduk Veljkova 3, 21000 Novi Sad, Serbia; milatstevan@gmail.com

**Keywords:** Poseidon criteria, ovarian reserve, ovarian stimulation, oocyte number, ovarian response

## Abstract

**Background/Objectives:** Poor responder patients represent the greatest challenge in ART. An inadequate response to COS strongly correlates with a reduced chance of conception. A novel classification of poor responders overcame a deficiency in the Bologna criteria and distinguished an expected and unexpected low ovarian response, allowing for an individual treatment approach to be created. In this study, we compared the clinical outcomes in poor responders, according to two different ovarian stimulation protocols, GnRH agonists and antagonists, classified according to the Poseidon criteria, to determine the most effective protocol for each group. **Methods:** This retrospective study involved 1323 low-prognosis women ranked according to the Poseidon classification and a control group of normal responders. **Results:** The GnRH-antagonist protocol showed some advantage in the Poseidon 1b group whereas the GnRH-agonist protocol was more effective in the Poseidon 4 group. There were no differences in live births or miscarriage rates in poor responders among these two protocols. **Conclusions:** Using both the agonist/antagonist approaches, live birth rates are two or even three times less in Poseidon patients in comparison to normal responders. The number of obtained oocytes, their maturity and quality, and women’s ages were found to be the most influential determinants for a successful outcome. Further investigations into ovarian stimulation strategies are required to enhance oocyte number and live birth occurrence.

## 1. Introduction

The inevitable component of assisted reproductive technologies (ARTs) is controlled ovarian stimulation (COS), which aims to recruit multiple oocytes and embryos to achieve conception [1]. According to evidence, between 10 and 15 oocytes are estimated to be the optimal response to ovarian stimulation [2]. Despite great advances in ART, some patients respond poorly to treatment [3]. There have been numerous publications on poor ovarian response (POR), and possible strategies to manage these patients have been proposed; however, no particular intervention has been adopted as a standard for enhancing outcomes [4].

POR is recognized as the retrieval of a low number of oocytes and consequently, less likelihood for conception and a higher risk of cycle cancelation [5]. The incidence of POR ranges between 9 and 24% [6]. Several etiologies of POR are well known. In general, defects in follicle-stimulating hormone (FSH) receptors as well as depleted ovarian reserve (OR), especially age-related, are the most common perpetrators for an adverse outcome [7]. Many authors emphasized the role of the various single-nucleotide polymorphisms (SNPs) in the FSH receptor (FSHR), especially ThR307AIa and Asn680Ser. The two polymorphisms are located in the same exonic region, and the presence of both SNPs causes a linkage disequilibrium between them and hurts the functionality of the FSHR. Particularly, the Asn680Ser polymorphism was found to cause reduced ovarian response to the exogenous gonadotropins during COS, leading to the unexpected lower oocyte number [8]. According to a meta-analysis in 2015, the Asn680Ser polymorphism could be a significant indicator for predicting the number of retrieved oocytes and poor ovarian response [9], while a recent meta-analysis in 2023 found no strong evidence of one gene examination about the improvement of COS outcomes and suggested polygenic analysis of different polymorphisms [10].

The definition of poor responder has been primarily established by the Bologna criteria [11]; still, the heterogeneity among patients has caused criticism regarding its clinical effectiveness. According to the Bologna criteria, young women with low Anti-Müllerian hormone (AMH) and older women with a normal AMH, both previously with a POR outcome, have similar chances for live births and should be managed in the same way [12]. However, research has shown that women in their 30s, even after retrieving a low number of mature oocytes, have a reasonable live birth rate (LBR), while women aged ≥40, with a low number of mature oocytes, have a two times lesser chance for a live birth [13,14]. Those differences are the repercussions of aneuploidy in oocytes that increases with age [15]. Moreover, the Bologna criteria could not distinguish patients with a low oocyte number obtained due to reduced ovarian reserve (OR) and patients with normal OR but inadequate oocyte number due to an impaired response to the gonadotropin stimulation [12].

Consequently, an analysis of poor responder patients’ medical etiology is inevitable since they require different clinical strategies pertaining to their age and AMH levels [16]. Hence, in 2016, the Poseidon (Patient-Oriented Strategies Encompassing IndividualizeD Oocyte Number) group proposed new criteria for poor responders based on age, antral follicle count (AFC), AMH, and response to a previous ovarian stimulation. According to these criteria, patients are stratified into four groups. The patients with an adequate OR and an unexpectedly low ovarian response are classified into Poseidon groups P1 and P2, and both groups are further stratified into subgroups (1a, 1b, 2a, and 2b) according to the number of retrieved oocytes. The patients with an expected low ovarian response caused by impaired OR are classified into P3 and P4 groups [6].

Various treatment regimens have been introduced for women belonging to Poseidon groups. Gonadotropin-releasing hormone agonist (GnRH-a) and GnRH antagonist (GnRH-ant) treatment is an important part of COS for many patients. The effectiveness of these protocols for POR patients remains unknown. Some researchers have found that the GnRH-ant protocol contributed to a higher oocyte number and LBR than the agonist protocol [17], while others found the GnRH-a protocol more effective in terms of fewer canceled cycles and higher pregnancy rates [18], particularly for young PORs [19]. It has also been shown that the early-follicular-phase long-acting GnRH-a long protocol was associated with higher LBR than the mid-luteal-phase short-acting GnRH-a long and GnRH-ant protocols [20]. Some studies demonstrate that the GnRH-ant protocol has yielded a greater number of mature oocytes, but this did not lead to an increase in LBRs in the Poseidon 3 and 4 groups [21]. An update of the Cochrane review has shown no evidence of a difference in LBRs between the GnRH-ant and the long GnRH-a protocols; however, the GnRH-ant treatment was associated with a lower incidence of any grade of ovarian hyperstimulation syndrome in comparison to the GnRH-a protocol [22]. Recently, the ESHRE (European Society for Human Reproduction and Embryology) and Reproductive Endocrinology Guideline Group declared Guidelines for POR, indicating an equal recommendation of GnRH-ant and GnRH-a since there was no difference in treating POR patients between the GnRH-a and the GnRH-ant ovarian stimulation [23]. Besides the conventional COS, modified natural, and mild protocols, flare-up and dual-stimulation protocols have been developed to enforce oocyte number and LBR occurrence [24,25,26] with no evidence to support a superior strategy. Nonetheless, longer ovarian stimulation, higher gonadotrophin doses, and adjuvant addition have been proposed, but they require further affirmation and investigation [2].

Most available evidence, as to the standard COS agonist and antagonist protocols supremacy, has been derived from the analysis of the Bologna criteria oriented towards poor responders while investigations concerning more homogeneous patient assembly may still be insufficient. Therefore, the purpose of this study is to investigate in vitro fertilization (IVF) outcomes between the GnRH-a and the GnRH-ant stimulation among Poseidon groups and subgroups and control groups to identify the most effective approach to a particular Poseidon group.

## 2. Materials and Methods

### 2.1. Patient Classification

This study, based on retrospective medical data, was conducted at the Ferona Fertility Clinic between January 2020 and January 2023 with the Ferona Fertility Clinic’s Ethics Board approval (No 1220-1/2-23) and the University of Novi Sad, Serbia, Faculty of Sciences Ethics Commission’s consent (No 0601-91/24-44), and it was in accordance with the Helsinki declaration. All women included in the study received one ovarian stimulation cycle and underwent fresh embryo transfer or subsequent frozen embryo transfer of cryopreserved surplus embryos from the fresh cycle, while patients who underwent the freezing of all embryos, genetic analysis, and oocyte donation were excluded. Furthermore, patients with no mature (metaphase II–MII) oocytes or developed embryos, canceled cycles, polycystic ovarian syndrome (PCOS), severe male infertility (no motile sperm, surgically retrieved sperm, or ≤4% of normal sperm morphology according to the Krüger criteria [27]), and untreated deleterious gynecological conditions such as severe endometriosis, hydrosalpinx, septum, myomas, and polyps were also not considered.

All patients were stratified according to the previously described Poseidon criteria (Table 1) [16]. The non-Poseidon group was introduced as a control group and comprised young (<35 years of age) normal responders with an adequate OR (≥1.21 ng/mL AMH; ≥10 oocytes). After their classification into the Poseidon groups and subgroups, patients were further stratified within a particular group by the received COS treatment (agonist or antagonist protocol).

### 2.2. Ovarian Stimulation and IVF Procedure

Ovarian stimulation protocols for eligible patients included the long GnRH-a and flexible GnRH-ant protocols with human menopausal gonadotropin (hMG), recombinant FSH (rec FSH), or hMG+ rec FSH. GnRH-a and GnRH-ant downregulations were applied according to the previously described proceedings [28]. Patients received an initial gonadotropin dose ranging from 75 to 300 IU, and doses were adjusted as needed according to the ovarian response. In the P4 group, apart from the standard GnRH antagonist stimulation protocol, an adjuvant letrozole/clomiphene citrate was added to one hundred and twenty-seven patients. Ovulation was initiated using recombinant human chorionic gonadotrophin (hCG) when appropriate estradiol (E2) level and at least one dominant follicle ≥18 mm was detected. Oocytes were retrieved 36 h after ovulation trigger and mature oocytes (metaphase II-MII) were fertilized via intracytoplasmic sperm injection. Embryo transfer (ET) was performed on Day 3 or Day 5, while surplus embryos were vitrified at the blastocyst stage. Good oocyte quality was assessed by the presence of a homogeneous and clear cytoplasm, of normal size and shape, a normal size and shape of the zona pellucida, an absence of dimorphism in the perivitelline space, and a normal size and shape of the first polar body [29]. Embryo quality was graded in relation to a previously published assessment [30]. ET or frozen embryo transfer (FET) was performed under ultrasound guidance, and vaginal/oral progesterone was provided for luteal support. FET cycles were performed under hormone replacement with endometrial preparations. Pregnancy was monitored with an increased serum βhCG level and a fetal heartbeat at the 6th gestational week.

### 2.3. Main Outcome Analysis

The main outcomes were analyzed, and we considered the number of retrieved, MII, good-quality, and fertilized oocytes; the number of developed and good-quality embryos; the number of developed blastocysts; the positive β-hCG rate; the clinical pregnancy rate (CPR); the live birth rate (LBR); and the miscarriage rate (MR), as well as the cumulative β-hCG, the cumulative CPR (CCPR), the cumulative LBR (CLBR), and the cumulative MR (CMR).

### 2.4. Statistical Analysis

Statistical analysis was performed using the SSPS software version 25.0. Differences in the main outcomes were identified using the ANOVA and Pearson’s chi-squared test, where a *p*-value of 0.05 or lower was considered statistically significant. Univariate and multivariate logistic regressions were applied to determine the predictive variables of the live birth. Using the G* power calculator (version 3.1), the sample size calculation was performed and the power analysis indicated that each group (control and Poseidon groups) required a minimum of 128 participants to achieve a statistical power of at least 0.80, with an error rate below 0.05 when comparing the two types of ovarian stimulation and assuming a minimum medium effect size of 0.25. However, in the P1a and P2a groups, the number of participants was less than 128.

## 3. Results

A total of 1323 women were included in the study. All included patients had one ovarian stimulation cycle, and the statistical analysis was based on the number of patients. For the patients who had cryopreservation of the surplus embryos after fresh ET and FET, of those embryos in the next cycle, the pregnancy/miscarriage outcomes were estimated as cumulative rates (fresh pregnancies/miscarriages + FET pregnancies/miscarriages).

The baseline and endocrine characteristics of the patients are presented in Table 2. The most frequent group was the P4 group. Among different groups, there were various infertility causes, with only endometriosis being equally widespread across all groups. Among all analyzed groups, sperm quality was generally good. Women in P4 had the highest FSH and luteinizing hormone (LH) levels. Endometrial thickness was appropriate among all groups, yet the thinnest endometrium was identified within patients belonging to P4 whereas patients in the NP group had the thickest endometrium.

An investigation of the COS among particular Poseidon groups and the control group is presented in Table 3a,b and was analyzed using ANOVA and a chi-square test. The GnRH-ant protocol was more frequently used compared to the GnRH-a protocol (*p* < 0.01). There was no difference in the clinical outcomes between the GnRH-a and the GnRH-ant protocols in NP, P1a, P2a, and P2b groups, whereas the GnRH-ant protocol was associated with a higher number of developed blastocysts (*p* < 0.05) and a positive b-hCG rate in the P1b group (Table 3a). Table 3b illustrates a comparison of the GnRH-a and the GnRH-ant protocols in the P3 and the P4 groups. In the P3 group, the GnRH-ant protocol was associated with a higher number of developed embryos (*p* < 0.05) and a higher number of transferred embryos (*p* < 0.05). In the P4 group, the GnRH-a protocol was associated with a higher number of retrieved oocytes (*p* < 0.01), a higher number of MII oocytes (*p* < 0.05), a higher number of good-quality oocytes (*p* < 0.05), a higher number of fertilized oocytes (*p* < 0.05), a higher number of developed embryos (*p* < 0.05), a higher number of transferable embryos (*p* < 0.05) and a higher number of developed blastocysts (*p* < 0.05) in comparison to the GnRH-ant protocol. However, in the P3 and P4 groups, there was no difference in the CPR, LBR, MR, or cumulative rates between the GnRH-a and GnRH-ant protocols.

A univariate logistic regression analysis (Table 4a) was applied to all patient populations to investigate LBR prediction using the GnRH-a or the GnRH-ant stimulation. A significant difference was not discovered between the two protocols in LBR. A multivariate logistic regression analysis (Table 4b) was performed to identify the correlation between significant potential influential factors (basal FSH and E2 level, AMH level, women’s age, affiliation to a particular Poseidon group, number of mature and good-quality oocytes, and number of developed blastocysts) and LBRs according to two different stimulation protocols among the entire Poseidon population. A significant contribution of analyzed independent predicted factors to live births in the GnRH-a approach was not discovered. On the contrary, the analysis revealed, in the GnRH-ant regimen, important predictive variables related to live births. It showed that women with a basal FSH level < 15 mIU/mL (25%), AMH level > 1.2 ng/mL, women <35 years of age, and women with higher mature and good-quality oocyte numbers had a significantly higher chance of live birth. Compared to the P4 group, women in the P1a, P1b, P2b, and P3 groups had a higher live birth likelihood. The basal E2 level and the number of developed blastocysts did not show a correlation with live births. Other potentially influential factors such as a cause of infertility, body mass index, or embryo transfer difficulty, were not investigated in this analysis, since we did not consider that those issues could have a significant impact on the live birth probability.

## 4. Discussion

The objective of the presented subject research was to present IVF outcomes and the chances for live births in POR patients using different COS. The research has confirmed the concept that achieving a pregnancy is most likely for younger women with good ORs and a high oocyte number [31]. In accordance with previous reports, the most adverse outcomes were discovered in the group of advanced-age women with reduced OR [32]. Our findings correlate with the conclusion of others that an increased number of retrieved oocytes in younger patients is strongly associated with a higher number of mature and good-quality oocytes, as well as a higher number of developed embryos and blastocysts [33]. In addition, the number of available embryos for cryopreservation was higher in the NP group, thereby increasing CLBR [34]. This evidence has confirmed that a woman’s age and adequate ovarian response play a crucial role [35].

The identification of a more potent stimulation protocol in POR promotes the main finding that both approaches were similar among all investigated groups. Oocyte and embryo apprehension usually was not the primary feature in the majority of research on agonist and antagonist usage in POR. Numerous published comparisons of the two COS protocols consider the effectiveness of ovarian stimulation through achieving pregnancy. In this research, in a patient with normal OR, there was no difference in the majority of the clinical outcomes, including CPR and LBR, between the GnRH-a and the GnRH-ant protocols. Other investigations have also shown no enhancement in LBR and CLBR in comparison to agonist and antagonist protocols [36,37,38]. The early-stage meta-analysis on pregnancy outcomes demonstrates that in patients with normal OR, the GnRH-a protocol showed an advantage in some clinical parameters over GnRH-ant, but both protocols were equally effective in CPR, LBR, and MR [39]. In addition, in our research, the GnRH-a and the GnRH-ant protocols did not show an advantage in CPR, LBR, and MR in patients with normal OR and an unexpectedly low response to stimulation regardless of the patient’s age. The further stratification of P1 and P2 into two subgroups according to the number of retrieved oocytes (P1a and P2a groups had <4 oocytes; P1b and P2b had 4–9 oocytes) demonstrate that neither the GnRH-a nor the GnRH-ant protocol showed any obvious advantage in terms of clinical outcomes such as CPR, LBR, or MR.

Contrary to these findings, some disclosures asserted agonist utilization for achieving higher LBR in younger [18,20] and older Poseidon groups [11]. It has been shown that early-follicular-phase long-acting GnRH-a long protocol (EFLL), a protocol developed by Chinese clinicians, was more effective in terms of CPR and LBR than the mid-luteal-phase short-acting GnRH-a long protocol or the GnRH-ant protocol in Poseidon group 3 [20]. The suggested explanation for those outcomes could be found in impaired endometrial receptivity using the EFLL protocol or the GnRH antagonists [20,40], while our results showed no correlation.

Regarding oocyte and embryo yield, some researchers emphasized antagonist utilization for producing a higher number of retrieved, mature, good-quality, and fertilized oocytes in some Poseidon groups [17,21]. Our results support evidence of equal efficiency with the GnRH-a and the GnRH-ant application in Poseidon groups 1, 2, and 3 [41,42,43]. In terms of embryo characteristics, except the P1b—where the GnRH-ant protocol was more effective in the blastocyst development and the positive ß-hCG rate, which did not result in higher CPR or LBR—we found no difference between the two GnRH downregulations in the number of developed and good-quality embryos and blastocysts in patients with normal OR, thereby supporting previous conclusions [37,44]. Considering that the P1b group consists of younger patients with normal ovarian reserve but producing suboptimal oocyte counts, we assumed that a higher blastocyst rate was achieved as a consequence of the patient’s young age and lower aneuploidy rates [45]. However, given that better blastocyst development did not result in higher clinical pregnancy and live birth rates, we supposed that gonadotropin dose might have influenced the endometrial receptivity in this group [46]. On the other hand, the number of patients in the P1b group who received the GnRH agonist approach was very low, so the significant difference in the outcome between the two protocols in the P1b group could have appeared as a consequence of the limited sample size.

The analysis of the effectiveness of the GnRH-a and GnRH-ant protocols conducted in the P3 and the P4 groups revealed an equal efficacy of both protocols in the P3 group, whereas, in the P4 group, we found the preferable influence of agonist protocol for oocyte and embryo yield. The long agonist protocol has been associated with a higher number of retrieved and mature oocytes and lower cancelation rates [47] due to more aligned follicular development [24]. Patients with elevated serum FSH levels may benefit from the long agonist protocol since they could experience the early growth of the leading follicle and suppress the development of the residing follicles [47], and the P4 group consisted precisely of advanced-age patients with the highest serum FSH levels. The number of developed embryos and blastocysts was also higher with the agonist protocol in the P4 group in support of the findings of Huang et al., 2018 [19], and Adel et al., 2021 [48], and contrary to other investigations where the antagonist protocol resulted in a higher or similar blastocyst yield [49]. Biological credibility could be found in a relationship between aneuploidy and blastocyst development given that aneuploidy embryos have a lower blastulation rate [50]. Although the majority of studies found no correlation between COS type and aneuploidy rate, Cascales et al., 2021, demonstrated higher aneuploidy rates in patients receiving a faster stimulation protocol, which had a detrimental effect on meiotic completion [51]. Since the agonist protocol requires a longer stimulation, we assume this discovery supports our results obtained with the agonist protocol. However, the aforementioned results should be carefully interpreted due to the low number of P4 patients who have received the GnRH agonist stimulation protocol.

In the present study, we found no relationship between LBR prediction and different stimulation protocols in all patient populations, according to univariate regression analysis. The lack of superiority of different protocols (the long agonist, antagonist, double-stimulation, and mini-flare) in LBR was also shown in the study of Mashayekhi et al., 2021 [52]. The multivariate logistic regression analysis of some potential predictive factors related to an LBR found differences when the GnRH-ant protocol was applied, while no divergences were identified in the GnRH-a approach. Women’s age, FSH, and AMH levels; the number of MII and good-quality oocytes; and certain Poseidon groups are significant predictive variables. Women aged <35 with a basal FSH level < 15 mIU/mL and an AMH level > 1.2 ng/mL; >4 mature and good-quality oocytes; and P1a, P1b, P2b, and P3 groups have enhanced likelihood for achieving pregnancy than women with adverse variables.

Given that we found no improvement as a result of the particular stimulation protocol in terms of ovarian response and pregnancy outcomes and that the chance of a live birth could be predicted using influential factors in the GnRH-ant stimulation, the GnRH-ant protocol could therefore be preferable due to its cost-effectiveness and shorter stimulation [53]. Our analysis has highlighted that only the P4 group may benefit from the GnRH-a protocol in oocyte and embryo pool increase. Although LBR did not differ significantly for older poor response women in the P4 group after the GnRH-a protocol, a higher oocyte yield and a generation of a large number of embryos for cryopreservation were required for optimal LBR. Numerous studies have proven that a higher number of blastocysts strongly correlates with live birth occurrence [54,55]. However, the obtained results should be cautiously interpreted due to the lower number of patients who received agonist stimulation and the restricted number of reports that justify those outcomes.

The quantity of published data on an investigation of compared IVF outcomes between the agonist and antagonist COS, among Poseidon groups and subgroups, is limited. Owing to a small sample size, patients are usually divided into Poseidon groups 1, 2, 3, and 4 without stratification into subgroups. In addition, investigations into COS are commonly intended for groups with the most adverse expected outcomes (P3 and P4). Therefore, an advantage of this particular research lies in a detailed analysis of outcomes among Poseidon groups and subgroups, while the obtained results could be useful in predicting the chance for live births and tailoring a treatment approach. Similarly to other publications, this study also has several limitations mainly due to its retrospective nature. Another weakness relates to the different doses and types of gonadotropin the patients received, as well as the administration of letrozole/clomiphene citrate to the older P4 patients with a very low ovarian reserve. According to the research, the effect of letrozole/clomiphene citrate is inconclusive, and we assume that in this study, adding letrozole/clomiphene citrate had no impact on the IVF outcomes since the P4 group was found to be the poorest among the Poseidon groups. The potential influence of these agents on the outcomes should be taken into consideration.

Finally, an analysis involved a low number of patients in some groups and subgroups. The calculated number of participants to be recruited was at least 128 per group. However, in the P1a and P2a groups, the number of participants was less than 128, so the absence of statistical difference could appear due to a low statistical power. Given that the nature of this study was retrospective, a required participant number in this group could not be achieved.

## 5. Conclusions

Live birth rates using both the agonist/antagonist approaches are two or even three times less in Poseidon patients compared to normal responders. GnRH agonist LBR in normal responders (control group) was 59.1% versus 20.0% in P1a, 26.9% in P1b, 0.0% in P2a, 17.9% in P2b, 22.7% in P3, and 12.2% in P4 groups while GnRH antagonist LBR in normal responders was 51.0% versus 27.47% in P1a, 33.02% in P1b, 11.43% in P2a, 23.45% in P2b, 24.4% in P3, and 9.06% in P4 groups. Aside from COS, a woman’s age, the number of oocytes, and their maturity and quality also determine pregnancy outcomes. The presented results indicate the variations among the poor responder population; therefore, the Poseidon classification could be a helpful managing tool for an individual treatment approach. Considering that this analysis has not established the most beneficial stimulation approach for all Poseidon patients, additional investigations are required to consolidate the supreme protocol for maximizing the oocyte number and LBR.

## Figures and Tables

**Table 1:** Poseidon classification of the poor responder patients into groups and subgroups according to their age, AMH, AFC, and ovarian response (the number of retrieved oocytes).

Characteristics of the Poseidon 1a Patient’s Group (P1a)	Characteristics of the Poseidon 1b Patient’s Group (P1b)	Characteristics of the Poseidon 2a Patient’s Group (P2a)	Characteristics of the Poseidon 2b Patient’s Group (P2b)	Characteristics of the Poseidon 3 Patient’s Group (P3)	Characteristics of the Poseidon 4 Patient’s Group (P4)
<35 years old	<35 years old	>35 years old	>35 years old	<35 years old	>35 years old
AFC ≥ 5	AFC ≥ 5	AFC ≥ 5	AFC ≥ 5	AFC < 5	AFC < 5
AMH ≥ 1.2 ng/mL	AMH ≥ 1.2 ng/mL	AMH ≥ 1.2 ng/mL	AMH ≥ 1.2 ng/mL	AMH < 1.2 ng/mL	AMH < 1.2 ng/mL
<4 oocytes retrieved	4–9 oocytes retrieved	<4 oocytes retrieved	4–9 oocytes retrieved	/	/

**Table 2:** Baseline and endocrine characteristics of Poseidon and non-Poseidon patients.

	Non-Poseidon (NP)	Poseidon Ia (PIa)	Poseidon Ib (PIb)	Poseidon IIa (PIIa)	Poseidon IIb (PIIb)	Poseidon III (PIII)	Poseidon IV (PIV)	χ/F	*p*
Total number	201	101	245	106	174	153	343	277.70	<0.001
Female age	29.45 ± 3.38	30.21 ± 3.49	29.77 ± 2.55	37.99 ± 3.54	38.78 ± 2.70	31.45 ± 2.26	40.86 ± 2.99	589.7	<0.001
Infertility cause, *n* (%)									
Unexplained	53 (26.00)	23 (22.80)	57 (23.30)	18 (22.50)	40 (23.00)	0 (0.00)	2 (0.60)	74.91	<0.001
Male factor	54 (27.50))	17 (16.80)	67 (27.30)	20 (25.00)	34 (19.50)	5 (3.30)	3 (0.90)	86.43	<0.001
Combined	32 (16.20)	18 (17.80)	62 (25.30)	17 (21.30)	52 (29.90)	63 (41.30)	135 (39.40)	191.62	<0.001
Ovulatory	7 (3.40)	6 (5.90)	4 (1.60)	5 (6.30)	3 (1.70)	49 (32.0%)	98 (28.60)	334.90	<0.001
Endometriosis	1 (0.50)	4 (4.00)	5 (2.00)	2 (2.50)	1 (0.60)	3 (2.00)	1 (0.30)	6.75	0.35
Tubal	24 (11.80)	14(13.90)	19 (7.80)	3 (3.80)	9 (5.20)	2 (1.30)	1(0.30)	32.0	<0.001
Uterine	22 (10.80)	8 (7.90)	22 (9.00)	10 (12.50)	28 (16.10)	1 (0.70)	4 (1.20)	44.87	<0.001
Multiple female causes	8 (3.90)	11(10.90)	9 (3.70)	5 (6.30)	7 (4.00)	28 (18.70)	99 (28.90)	295.52	<0.001
Sperm quality, *n* (%)									
Low	76 (38.77)	28 (22.72)	101 (41.22)	43 (28.70)	55 (31.61)	64 (28.23)	86 (25.07)	83.96	<0.001
Good	125 (61.23)	73 (77.28)	144 (58.78)	107 (71.30)	119 (68.39)	89 (71.77)	257 (75.93)	224.65	<0.001
Endocrine profile									
AMH level ng/mL	3.75 ± 2.18	3.15 ± 2.57	3.28 ± 2.10	2.25 ± 1.59	2.21 ± 1.16	0.68 ± 0.32	0.49 ± 0.29	134.55	<0.001
FSH level mIU/mL	6.48 ± 1.86	8.07 ± 2.74	7.27 ± 2.40	8.43 ± 3.59	7.79 ± 2.40	9.37 ± 4.96	11.79 ± 6.97	37.75	<0.001
LH level IU/mL	5.60 ± 2.79	6.12 ± 3.66	5.76 ± 3.07	5.96 ± 2.51	5.92 ± 3.25	5.45 ± 4.05	6.60 ± 4.21	2.75	0.03
E2 level pg/mL	78.14 ± 105.07	85.18 ± 80.24	85.65 ± 125.35	97.23 ± 125.17	91.64 ± 74.71	98.03 ± 129.76	99.57 ± 110.45	1.06	0.39
Endometrial thickness (mm)	11.63 ± 1.87	10.63 ± 1.99	11.48 ± 1.91	9.89 ± 1.73	10.48 ± 1.93	10.15 ± 2.05	9.27 ± 1.90	46.87	<0.001

*p*-value ≤ 0.05 is considered statistically significant; confidence intervals: 95% (*p* ≤ 0.05), 99% (*p* ≤ 0.01). Good sperm quality reference values: concentration > 15 million/mL, progressive motility (A + B) > 32%, normal sperm morphology > 4%; low sperm quality: lower estimated parameters compared to reference values.

**Table 3:** Clinical outcomes of GnRH long agonist and GnRH flexible antagonist ovarian stimulation protocols among control non-Poseidon group and Poseidon groups and subgroups.

(a) Non-Poseidon and Poseidon 1a and 1b Groups																
	Non-Poseidon (NP)						Poseidon 1a (P1a)						Poseidon 1b (P1b)			
	GnRH a		GnRHant		F/χ	*p*	GnRH a		GnRHant	F/χ		*p*	GnRH a	GnRHant	F/χ	*p*
No. of patients (fresh cycles)	44		157 *		63.53	<0.01	10		91 *	64.96		<0.01	30	215 *	175.05	<0.01
No. of retrieved oocytes	12.84 ± 2.69		13.20 ± 3.34		0.65	NS	2.20 ± 0.79		2.44 ± 0.73	0.97		NS	6.73 ± 1.66	6.59 ± 1.67	−0.39	NS
No. of MII oocytes	8.57 ± 2.23		8.72 ± 3.20		0.24	NS	1.60 ± 0.70		1.82 ± 0.84	0.92		NS	4.35 ± 1.50	4.59 ± 1.77	0.69	NS
No. of good-quality oocytes	6.95 ± 3.03		6.75 ± 4.20		−0.29	NS	1.50 ± 0.71		1.47 ± 0.97	−0.09		NS	3.08 ± 2.12	3.59 ± 2.33	1.07	NS
No. of fertilized oocytes	7.0 ± 2.12		7.11 ± 2.98		0.18	NS	1.60 ± 0.70		1.63 ± 0.73	0.11		NS	3.27 ± 1.19	3.68 ± 1.58	1.27	NS
No. of developed embryos	6.89 ± 2.13		6.75 ± 3.03		−0.09	NS	1.60 ± 0.70		1.62 ± 0.73	0.06		NS	1.19 ± 1.23	3.52 ± 1.60	1.02	NS
No. of high-quality embryos	4.35 ± 2.26		4.16 ± 3.02		0.27	NS	1.33 ± 0.50		0.92 ± 0.74	−1.63		NS	1.62 ± 1.47	2.23 ± 1.63	1.85	NS
No. of transferred embryos	1.89 ± 0.46		1.81 ± 0.64		−0.53	NS	1.60 ± 0.70		1.47 ± 0.55	−0.71		NS	2.04 ± 0.60	1.89 ± 0.52	−1.33	NS
No. of developed blastocysts	3.36 ± 2.27		2.64 ± 2.12		−1.91	NS	0.00 ± 0.00		0.36 ± 0.51	0.98		NS	1.33 ± 1.73	2.40 ± 1.45 *	2.04	*p* < 0.05
Positive β-hCG rate (%)	47.7		46.5		0.02	NS	40.00		37.36	0.37		NS	23.1	37.44 *	2.09	*p* < 0.05
CPR (%)	47.7		46.5		0.02	NS	20.00		32.97	0.74		NS	23.1	36.21	1.78	NS
LBR (%)	47.7		43.9		0.19	NS	20.00		27.47	0.28		NS	23.1	33.02	0.48	NS
MR (%)	0.0		1.9		0.85	NS	0.00		1.1	0.11		NS	0.00	6.98	1.72	NS
CCPR (%)	61.4		54.8		0.61	NS	20.00		34.44	0.85		NS	26.9	39.51	1.57	NS
CLBR (%)	59.1		51.0		0.91	NS	20.00		27.47	0.35		NS	26.9	33.02	0.39	NS
CMR (%)	2.3		3.8		0.09	NS	0.00		1.1	0.11		NS	0.00	6.98	1.84	NS
**(b) Poseidon 2a and 2b, P3, and P4 Groups**																
	**Poseidon 2a (P2a)**				**Poseidon 2b (P2b)**				**Poseidon 3 (P3)**				**Poseidon 4 (P4)**			
	**GnRH a**	**GnRHant**	**F/χ**	***p***	**GnRH a**	**GnRHant**	**F/χ**	***p***	**GnRH a**	**GnRHant**	**F/χ**	***p***	**GnRH a**	**GnRHant**	**F/χ**	***p***
No. of patients (fresh cycles)	8	98 *	50.24	<0.01	38	136 *	50.58	<0.01	22	131 *	77.65	<0.01	25	318 *	252.75	<0.01
No. of retrieved oocytes	2.13 ± 0.99	2.29 ± 0.75	0.49	NS	6.33 ± 1.40	6.32 ± 1.57	−0.03	NS	4.91 ± 2.45	4.62 ± 2.52	−0.51	NS	5.19 ± 2.37 **	2.98 ± 2.01	−5.3	<0.01
No. of MII oocytes	1.63 ± 0.74	1.63 ± 0.72	0.03	NS	4.74 ± 1.48	4.40 ± 1.48	−1.27	NS	2.96 ± 1.53	3.21 ± 1.80	0.62	NS	3.54 ± 1.86 **	2.28 ± 1.50	−4.04	<0.01
No. of good-quality oocytes	1.25 ± 1.04	1.39 ± 0.86	0.35	NS	3.90 ± 2.10	3.40 ± 2.03	−1.33	NS	2.23 ± 1.48	2.54 ± 1.99	0.71	NS	2.88 ± 2.20 **	1.68 ± 1.56	−3.69	<0.01
No. of fertilized oocytes	1.38 ± 0.52	1.49 ± 0.65	0.44	NS	3.67 ± 1.63	3.61 ± 1.48	−0.22	NS	2.05 ± 0.95	2.62 ± 1.49	1.75	NS	2.96 ± 1.78 **	2.00 ± 1.24	−3.72	<0.01
No. of developed embryos	1.25 ± 0.46	1.44 ± 0.65	0.79	NS	3.59 ± 1.70	2.22 ± 0.63	−0.59	NS	1.96 ± 1.00	2.60 ± 1.49 *	1.94	*p* < 0.05	2.77 ± 1.70 **	1.93 ± 1.22	−3.33	<0.01
No. of high-quality embryos	1.00 ± 0.53	0.94 ± 0.80	−0.24	NS	1.72 ± 1.72	2.08 ± 1.44	1.33	NS	1.18 ± 1.14	1.56 ± 1.23	1.36	NS	1.15 ± 1.16	1.10 ± 1.05	−0.29	NS
No. of transferred embryos	1.38 ± 0.74	1.40 ± 0.62	0.08	NS	2.26 ± 0.72	2.22 ± 0.63	−0.31	NS	1.50 ± 0.60	1.79 ± 0.53 *	2.29	*p* < 0.05	2.08 ± 0.80 **	1.66 ± 0.77	−2.71	<0.01
No. of developed blastocysts	0.00 ± 0.00	0.03 ± 0.25	0.32	NS	0.33 ± 1.15	0.23 ± 0.75	−0.63	NS	0.00 ± 0.00	0.82 ± 1.22	1.16	NS	0.27 ± 0.96 **	0.05 ± 0.36	−2.68	<0.01
Positive β-hCG rate (%)	12.5	21.43	0.33	NS	35.9	38.64	0.09	NS	27.3	29.8%	0.06	NS	24.00	15.63	1.14	NS
CPR (%)	0.00	17.14	1.59	NS	23.1	34.09	1.69	NS	22.7	27.5	0.22	NS	12.00	13.13	0.04	NS
LBR (%)	0.00	11.43	1.01	NS	17.9	23.45	0.53	NS	18.2	22.9	0.26	NS	8.2	9.06	0.05	NS
MR (%)	0.00	5.71	0.48	NS	5.1	10.61	1.07	NS	4.5	4.6	0.01	NS	3.8	4.06	0.01	NS
CCPR (%)	0.00	17.14	1.59	NS	25.6	36.36	1.54	NS	27.3	29.0	0.03	NS	16.00	13.75	0.08	NS
CLBR (%)	0.00	11.43	1.01	NS	17.9	23.45	1.01	NS	22.7	24.4	0.04	NS	12.2	9.06	0.11	NS
CMR (%)	0.00	5.71	0.48	NS	7.7	10.61	0.29	NS	4.5	4.6	0.01	NS	3.8	4.06	0.01	NS

Results were presented as means ± standard deviation or percentage; *p*-value ≤ 0.05 is considered statistically significant; confidence intervals: 95% (*p* ≤ 0.05), 99% (*p* ≤ 0.01); * GnRH antagonists > GnRH agonists, ** GnRH agonists > GnRH antagonists. Abbreviations: β-hCG -hCG (beta human chorionic gonadotropin); CPR (clinical pregnancy rate); LBR (live birth rate); MR (miscarriage rate); CCPR (cumulative clinical pregnancy rate); CLBR (cumulative live birth rate); CMR (cumulative miscarriage rate); CCPR, CLBR, and CMR are cumulative rates and present the outcomes of both fresh ET and subsequent FET after one ovarian stimulation cycle.

**Table 4:** (**a**) Univariate logistic regression of live birth prediction using GnRH-a and GnRH-ant stimulation protocol; (**b**) multivariate logistic regression of predictive variables related to live birth in GnRH-a and GnRH-ant stimulation protocols in Poseidon patients.

(a)							
	Odd Ratio	*p* Value			CI 95%		
GnRH-a	Reference group						
GnRH-ant	1.20	0.44			0.75–1.92		
**(b)**							
		**Gn RH-ant**			**GnRH-a**		
		**Odd**	***p***	**CI**	**Odd**	***p***	**CI**
Basal FSH (mIU/mL)	<15	0.25	<0.001	0.12–0.55	0.01	0.99	0.00–0.01
	>15	Reference group			Reference group		
AMH (ng/mL)	>1.2	0.43	<0.001	0.31–0.60	0.86	0.75	0.34–2.19
	<1.2	Reference group			Reference group		
Basal E2 (pg/mL)	<50	0.87	0.36	0.64–1.18	0.47	0.10	0.19–1.15
	>50	Reference group			Reference group		
Age	<35	0.38	<0.001	0.28–0.53	0.53	0.16	0.22–1.29
	>35	Reference group			Reference group		
Poseidon group	Ia	4.43	<0.001	2.82–6.98	2.70	0.19	0.61–11.93
	Ib	3.67	<0.001	2.05–6.58	1.83	0.55	0.25–13.06
	IIa	1.42	0.35	0.68–2.95	0.00	0.99	0.00–0.00
	IIb	3.04	<0.001	1.76–5.26	1.66	0.50	0.39–7.12
	III	2.94	<0.001	1.72–5.07	2.16	0.34	0.45–10.32
	IV	Reference group			Reference group		
No. of MII oocytes	>4	0.33	<0.001	0.27–0.55	0.44	0.10	0.16–1.17
	<4	Reference group			Reference group		
No. of good-quality oocytes	>4	0.38	<0.001	0.27–0.55	0.44	0.10	0.16–1.17
	<4	Reference group			Reference group		
No. of developed blastocysts	>1	0.79	0.58	0.33–1.84	0.16	0.22	0.01–2.98
	1	Reference group			Reference group		

*p*-value ≤ 0.05 is considered statistically significant; confidence intervals: 95% (*p* ≤ 0.05), 99% (*p* ≤ 0.01).

## Data Availability

All data involved in this work will be made available by the corresponding author upon request (kristina.pogrmic@dbe.uns.ac.rs).
